# Griscelli Syndrome: A Case Report from Pakistan, A Review of the Literature, and an Approach to Hematological Disorders Associated With Albinism

**DOI:** 10.7759/cureus.86445

**Published:** 2025-06-20

**Authors:** Maryam Afzal, Sobia Ashraf, Lubna Humayun, Raana Akhtar, Ayesha Masood, Shagufta Iram, Sehar Imran

**Affiliations:** 1 Pathology, University College of Medicine and Dentistry, The University of Lahore, Lahore, PAK

**Keywords:** chediak-higashi syndrome, griscelli syndrome, hemophagocytic lymphohistiocytosis (hlh), hermansky-pudlak syndrome, oculocutaneous albinism (oca)

## Abstract

Griscelli syndrome (GS) is a rare genetic disorder that is classified into three distinct types. Partial oculocutaneous albinism is common to all three types. In addition, neurological abnormalities and immunodeficiency are seen in types 1 and 2, respectively. Hemophagocytic lymphohistiocytosis (HLH) is common in GS-2. We present a case of a four-year-old boy who presented with features of albinism, recurrent infections, and hepatosplenomegaly. His complete blood count (CBC) revealed pancytopenia, and bone marrow biopsy showed prominent hemophagocytic activity. Serum ferritin was elevated, and fibrinogen was low. The diagnostic criteria for HLH were met. Hair shaft microscopy revealed large, irregularly spaced clumps of melanin in the medulla. A diagnosis of GS-2 was made. Unfortunately, the patient died before mutation analysis could be performed.

Along with this case report, we have included a literature review of 42 cases from 33 case reports and case series. We also propose a diagnostic approach to three hematological disorders associated with albinism, Chediak-Higashi syndrome (CHS), Hermansky-Pudlak syndrome (HPS), and GS.

## Introduction

Griscelli syndrome (GS) is a rare genetic disease. By the year 2020, almost 150 cases had been reported, with the Turkish and Mediterranean regions as the main contributors to the bulk of the disease [1]. The prevalence of the disease was found to be less than 1 in 1,000,000 [1]. The exact prevalence of this disease is not known mainly because of the rarity of the disease, overlapping symptoms with other syndromes, and the high likelihood of being missed due to improper and incomplete evaluation [2]. GS shows an autosomal recessive (AR) pattern of inheritance. As evident by the pattern of inheritance, the disease clusters are most commonly found in families with high rates of consanguinity. It was first described by Griscelli in 1978 [3]. Three types of GS have been defined based on symptoms and driver mutations. Partial oculocutaneous albinism is common to all three forms of the disease. Patients present with silver-gray hair and hypopigmentation of skin. This appearance results from pigmentary dilution in the skin and hair. These patients may have a normal number of melanosomes in the skin, but they have a reduced capacity for melanin production [4].

## Case presentation

A four-year-old boy presented with a history of an off-and-on drop in hemoglobin. His parents gave a history of recurrent ear and throat infections since infancy, and were concerned about his distended abdomen.

He was born via lower-segment cesarean section and cried immediately after birth. His birth weight was 3.2 kg. The mother denied any complications, fever, or infections during pregnancy. He achieved developmental milestones on time. His parents were unrelated. He had three siblings. Two sisters were alive and healthy. His younger brother had similar symptoms and died at nine months of age. He also had two second-degree male cousins with the same symptoms who died in the first decade of their lives. The female cousins in the same family were unaffected.

He was an alert, pale-looking child, well-oriented in time and space. His height and weight were normal for his age. We noticed, however, that he had silver-gray hair and eyebrows. The abdomen was distended with a palpable liver and spleen. No abnormality was detected in the systemic examination. Neurological and respiratory examinations, in particular, were normal. There was no history of bleeding or easy bruising.

His parents were particularly depressed because they had been to several medical practitioners. One of them had advised him to undergo hemoglobin electrophoresis under the suspicion of hemoglobinopathy. His hemoglobin electrophoresis was normal. At the time of presentation, he was referred to us for bone marrow aspirate and trephine biopsy under the suspicion of Gaucher’s disease.

We performed a complete blood count (CBC), which showed pancytopenia, i.e., a reduction in all three cell lines. The mean corpuscular volume (MCV) and mean corpuscular hemoglobin (MCH) were both reduced. White blood cells (WBC) had a majority of lymphocytes (80%). The details of the CBC are given in Table 1.

**Table 1:** Complete blood count (CBC) findings of the patient MCH: mean corpuscular hemoglobin, MCV: mean corpuscular volume, WBC: white blood cells

Parameter	Patient’s Results	Units	Interpretation
Hemoglobin	7.1	g/dL	Low
Hematocrit	23.1	%	Low
MCV	73	fL	Low
MCH	22.3	pg	Low
WBC count	3.8	x 10^9^/L	Normal
Neutrophils	14	%	Low
Lymphocytes	80	%	Raised
Monocytes	4	%	Normal
Eosinophils	2	%	Normal
Platelet count	46	x 10^9^/L	Low

In this case of partial albinism with hepatosplenomegaly and reduced blood counts, we had three differential diagnoses: Chediak-Higashi syndrome (CHS), Griscelli syndrome (GS), and Hermansky-Pudlak syndrome (HPS). Pancytopenia and no history of bleeding, however, made HPS less likely. Recurrent infections also favored CHS and GS. Next, we examined a peripheral blood film (PBF). The peripheral film confirmed reduced platelet count. Red blood cells (RBC) showed a dimorphic picture with two populations, normochromic normocytic and hypochromic microcytic. Reactive lymphocytes were noted. White blood cells (WBC) had a normal appearance. Giant granules in granulocytes and lymphocytes were particularly sought. No such abnormality was noted. This essentially ruled out CHS. No hemoparasite was seen on the smear.

**Figure 1:**
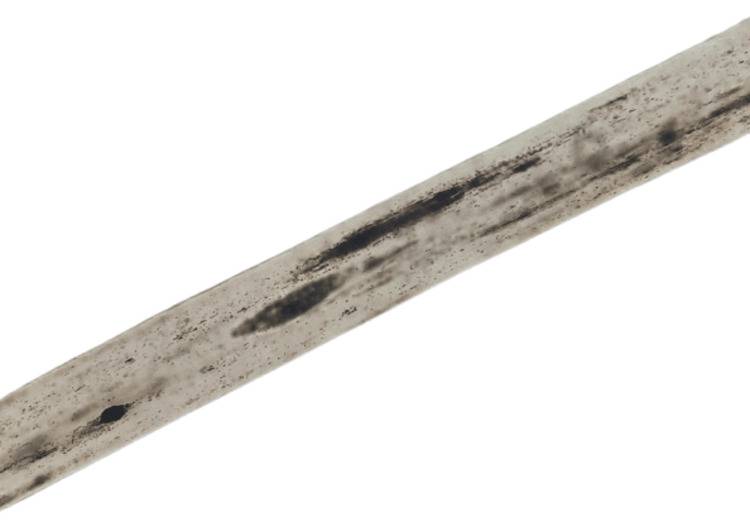
Hair shaft image of the patient Whole-mount microscopy of the hair shaft shows large, irregularly spaced clumps of melanin in the medulla.

Hair shaft microscopy was done on a binocular compound microscope. It revealed large, irregularly spaced clumps of melanin in the medulla. This appearance is also sometimes referred to as the “road-dividing-line” pattern. Hair shaft microscopy further confirmed it was not CHS, and the pattern was reported to be suggestive of GS. The microscopic appearance of the hair shaft is shown in Figure 1.

The patient had a very low platelet count. Bleeding time and platelet aggregation studies could not be performed. The patient’s parents had also negated any bleeding episodes in the patient or his affected sibling. This, along with a normal respiratory examination, made HPS less likely.

**Figure 2:**
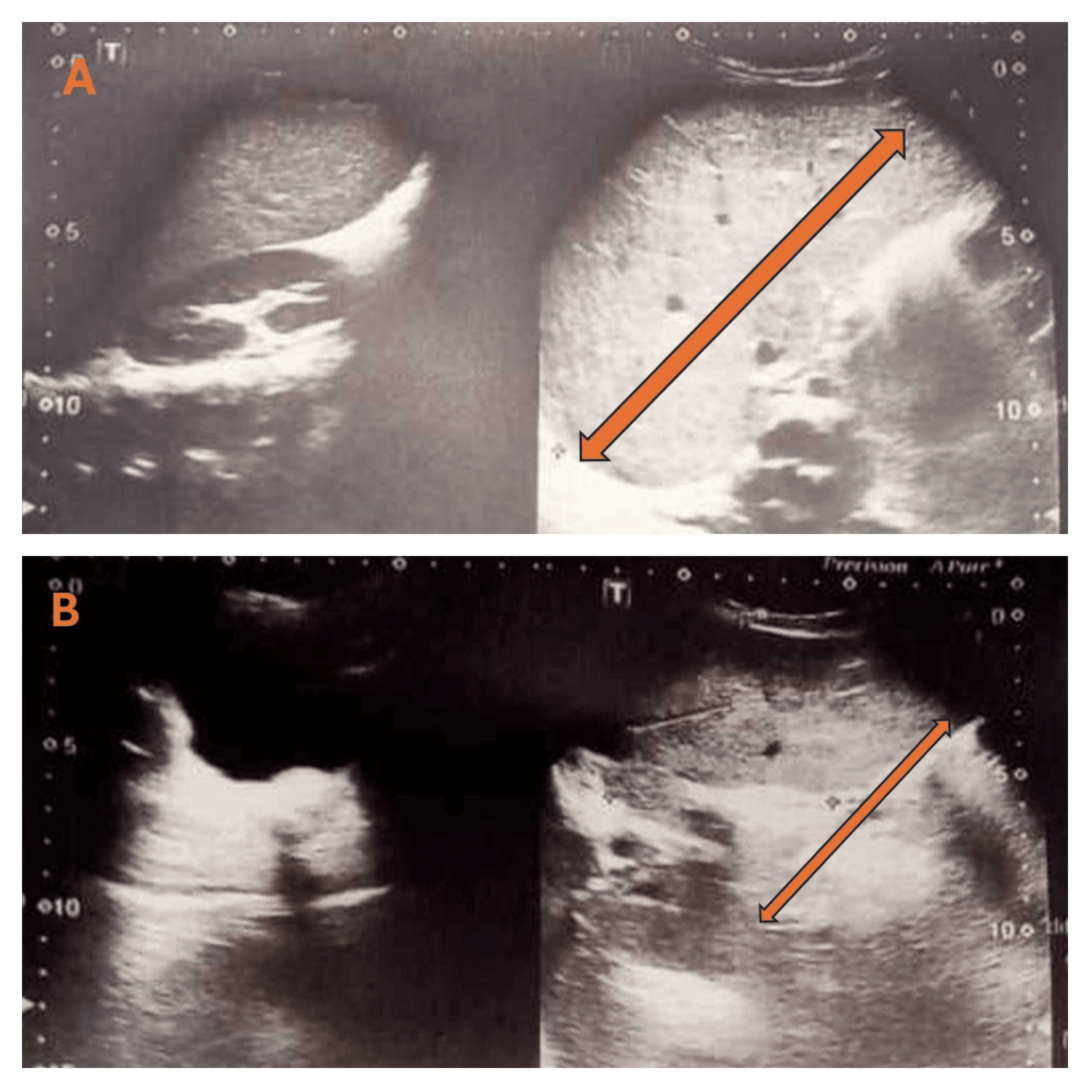
Ultrasound abdomen for organomegaly A: Enlarged liver; the arrow shows the size of the liver. B: Enlarged spleen; the arrow shows the size of the spleen.

Abdominal ultrasound was advised. It showed an enlarged liver and spleen. The liver was 15.6 cm in size with normal echotexture. The spleen was found to be enlarged with a splenic index of 80.52. The findings on ultrasound are shown in Figure 2.

**Figure 3:**
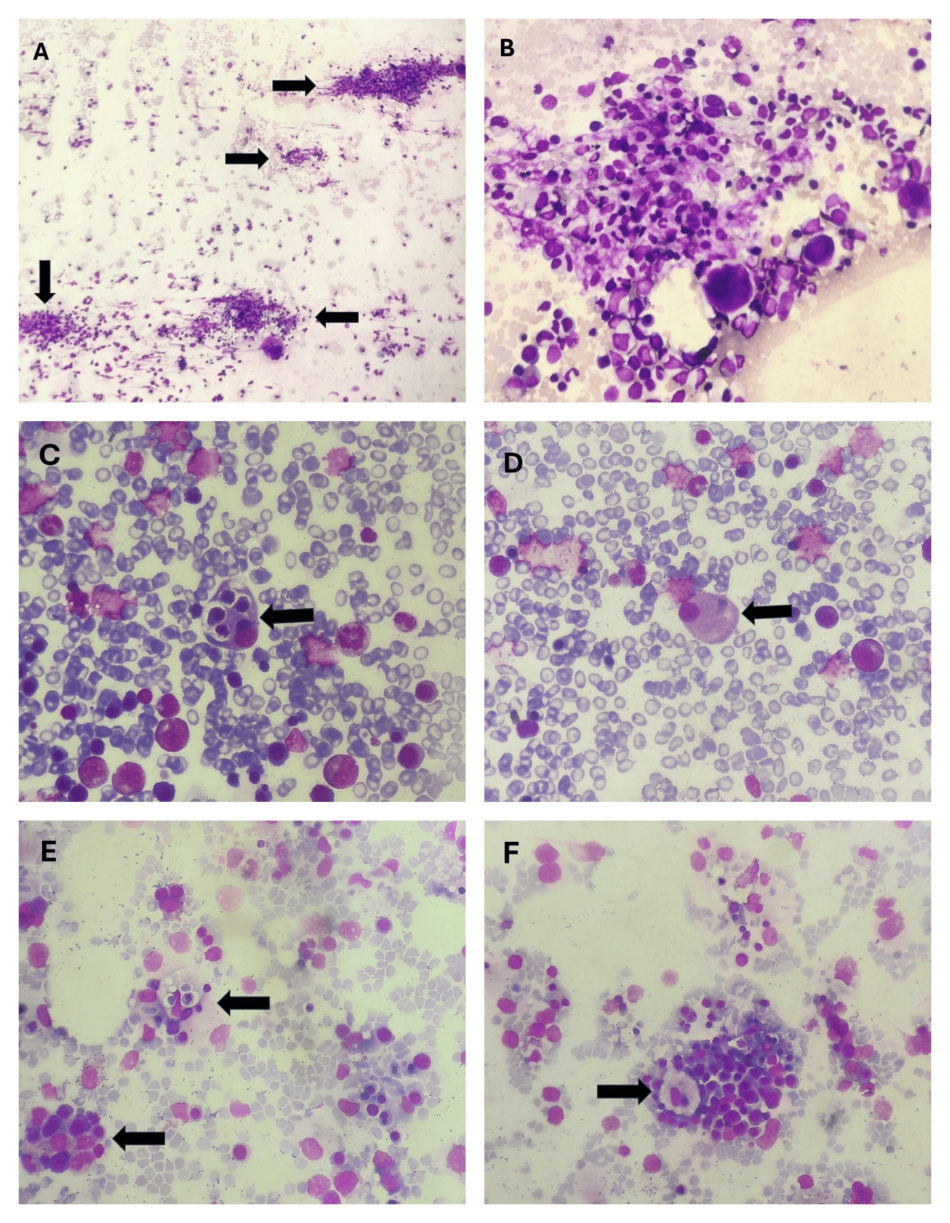
Bone marrow aspirate findings of the patient A: Arrows show hemophagocytosis. B: Magnified view of one of the macrophages in A. C-F: Arrows show macrophages engulfing hematopoietic cells.

**Figure 4:**
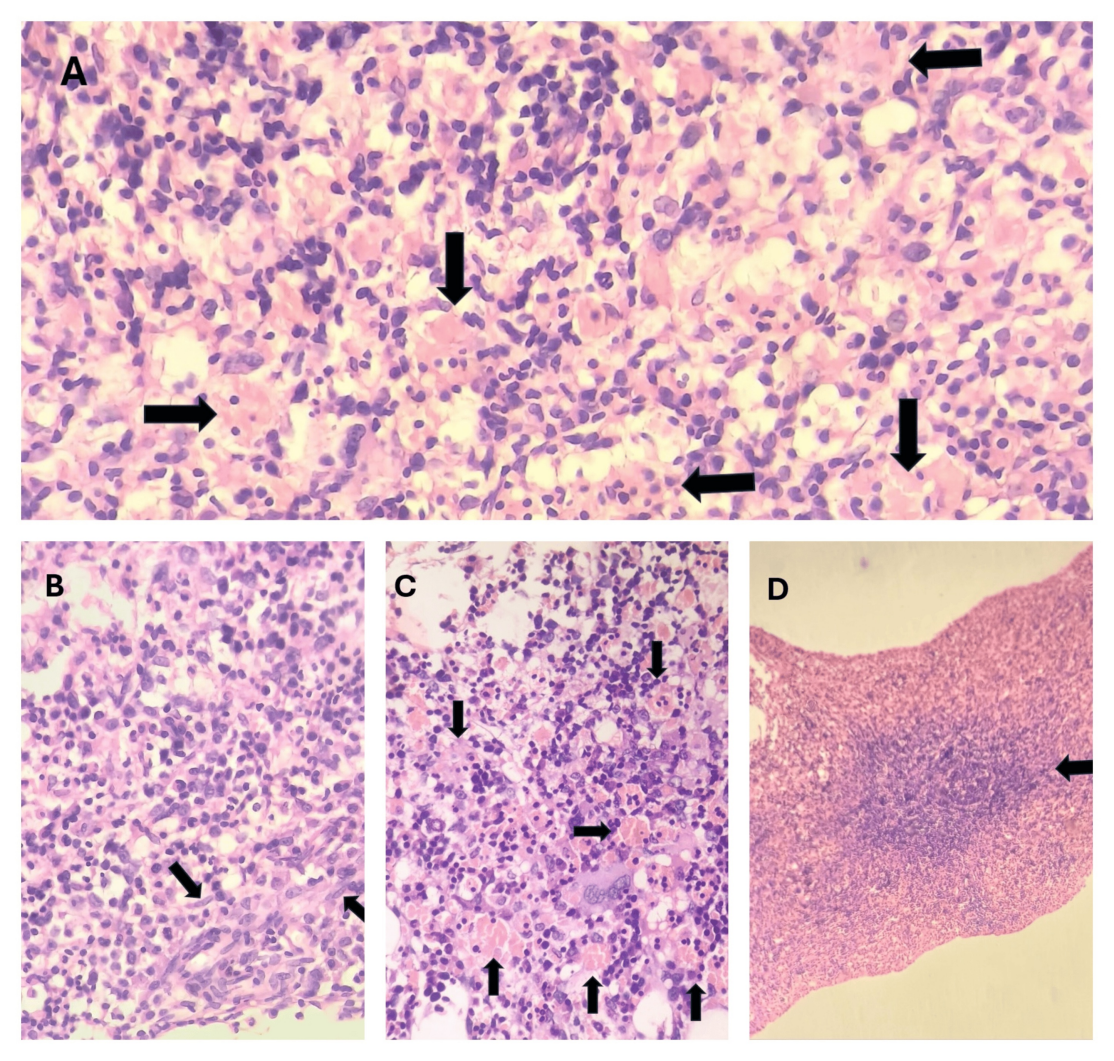
Trephine biopsy images of the patient A: Arrows show increased hemophagocytic activity. B: The image shows an ill-defined granuloma with arrows pointing toward epithelioid cells. C: Arrows show macrophages engulfing hematopoietic cells. D: The image shows nodule in the trephine biopsy section.

**Figure 5:**
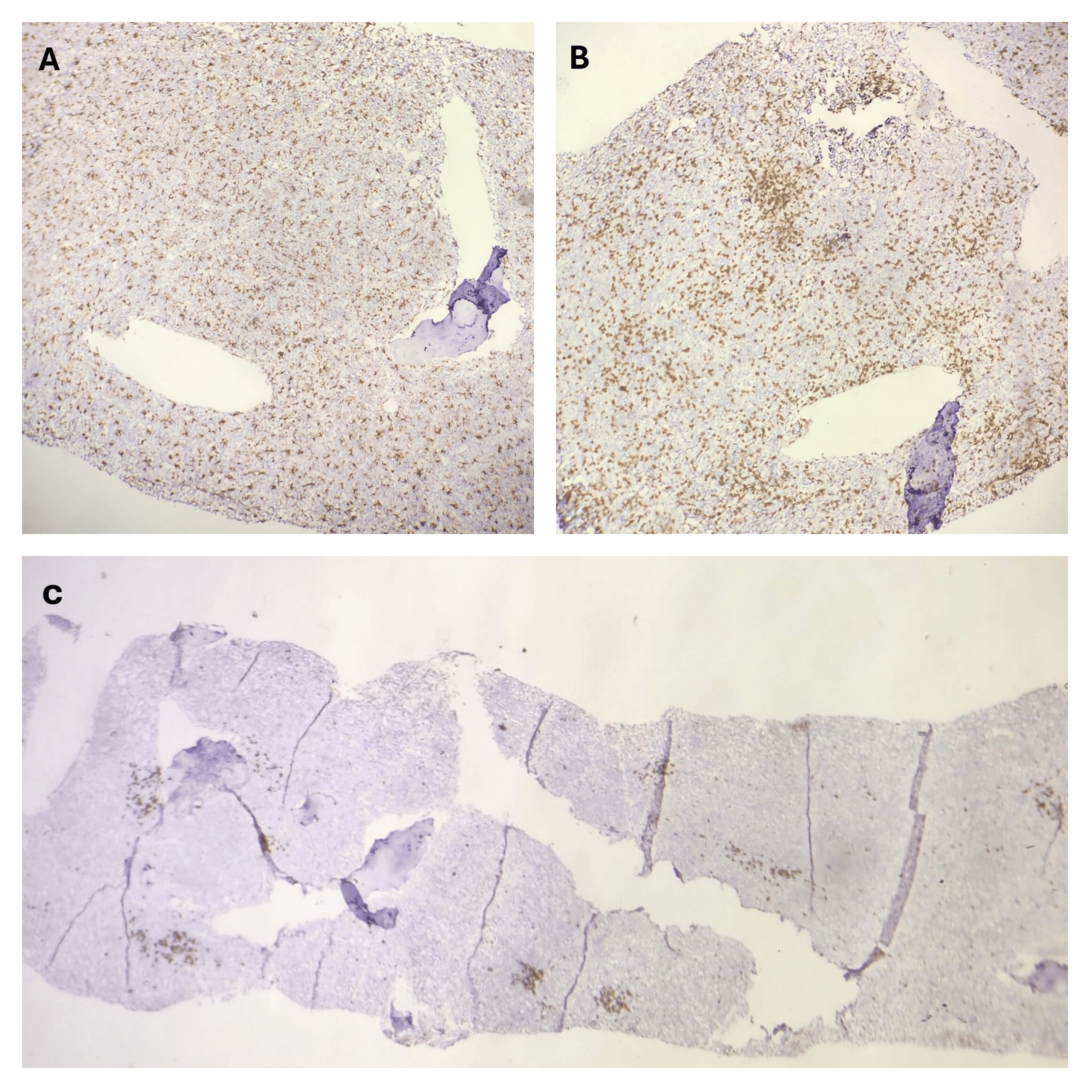
Immunohistochemistry on trephine biopsy A: CD68 - diffusely positive in the macrophages. B: CD3 - positive in reactive T lymphocytes. C: CD20 - positive in B lymphocytes. Overall, immunohistochemistry confirmed increased macrophage activity and reactive nature of the nodule.

Bone marrow aspirate and trephine biopsy (BMAT) were performed. An adequate aspirate and trephine specimen was effortlessly obtained. Bone marrow aspirate showed erythroid hyperplasia with a myeloid-to-erythroid ratio of 1:3 (normal: 2:1 to 12:1). Myelopoiesis was hypoplastic. Abnormal granulation was not seen in the myeloid lineage. Megakaryocytes were adequate in number. The blast percentage was less than 5%. Markedly increased hemophagocytic activity was noted on both bone marrow aspirate and trephine biopsy. Trephine biopsy also showed lymphoid nodules and multiple ill-defined granulomas without caseated necrosis. Immunohistochemistry (CD20 and CD3) revealed the lymphoid nodule to be reactive. CD68 was used for the confirmation of macrophages. It was diffusely positive throughout the trephine biopsy. Due to prominent hemophagocytosis, hepatosplenomegaly, and fever, we suspected hemophagocytic lymphohistiocytosis (HLH). Figures 3-4 show increased hemophagocytic activity in the bone marrow aspirate and trephine biopsy, respectively, and Figure 5 shows immunohistochemistry findings.

We requested serum ferritin and serum fibrinogen levels. Serum ferritin was raised, and serum fibrinogen was reduced. The patient also had raised serum bilirubin and liver enzymes. Five out of eight diagnostic criteria were fulfilled. The diagnosis of HLH was confirmed. The results of these investigations are shown in Table 2.

**Table 2:** Biochemical profile of the patient

Test	Patient’s Results	Units	Interpretation
Total bilirubin	1.3	mg/dL	Elevated
Direct bilirubin	0.9	mg/dL	Normal
Indirect bilirubin	0.4	mg/dL	Normal
Alanine aminotransferase (ALT)	88	u/L	Elevated
Aspartate aminotransferase (AST)	176	u/L	Elevated
Alkaline phosphatase (ALP)	2022	u/L	Elevated
Serum creatinine	0.3	mg/dL	Normal
Blood sugar (random)	95	mg/dL	Normal
Serum ferritin	11150	ng/mL	Elevated
Serum fibrinogen	78	mg/dL	Reduced

In summary, our patient had partial albinism with large, irregular clumps of melanin on the hair shaft, hepatosplenomegaly, and HLH. The diagnosis of Griscelli syndrome type 2 (GS-2) was made. Unfortunately, the patient died within days, and mutation analysis could not be done.

Our patient died within two weeks of his first encounter with us. His uncle (guardian) gave consent for the publication of the case report. All the images in the case report have been added with the prior permission of the patient's guardian.

## Discussion

Griscelli syndrome presents in the form of three distinct presentations. Thus, it is considered to have three types. Griscelli Syndrome Type 1 (GS-1) is characterized by partial albinism along with primary neurological complications. These patients often experience seizures, hypotonia, mental retardation, developmental delay, and developmental regression as CNS manifestations of the disease. GS-1 is caused by a mutation in the Myosin VA (MYO5A) gene [5]. Elejade syndrome also shows a similar presentation and driver mutations. There are divided opinions on whether GS-1 and Elejade syndrome are two names for one entity, or two different diseases impossible to differentiate from each other [6]. In Griscelli Syndrome Type 2 (GS-2), hypopigmentation is coupled to immunologic dysfunction. These patients have a history of recurrent fever and infections, particularly respiratory and skin infections. Reduced immunoglobulin levels are observed. GS-2 is the most lethal of all the types of GS because of its propensity toward HLH, which is thought to be the result of immune dysregulation. Most children succumb to death within the first decade of life. GS-2, although not associated with primary CNS abnormalities, can have neurological complications secondary to immune dysregulation and HLH. This is thought to be due to T-lymphocytes and macrophage infiltration of the CNS. The culprit mutation behind GS-2 is RAB27A [7]. Griscelli syndrome Type 3 (GS-3) presents with partial oculocutaneous albinism and large, irregularly spaced clumps of melanin on the hair shaft. Neurologic and immunologic abnormalities are not observed in this type of GS. The driver mutation lies in the Melanophilin (MLPH) gene [8].

Due to the rarity of this disease, most of the data in the literature are present in the form of case reports and letters to the editor. Some case series and very few research articles are available. We present data collected from 33 publications, which include 42 cases of GS [2,9-41]. Since our patient was diagnosed with GS-2, we focused more on the publications about this type of disease, and the collected data includes 24 cases of GS-2 [2,9-31]. We included nine cases of GS-1 and GS-3 each [32-40]. We collected information about demographics, disease type, presenting complaints, disease course, family history, examination findings, and investigations. For clarity, we have tabulated the case reports under the main headings of history, examination findings, investigations performed, and diagnosis. Cases with GS-2 are presented before GS-3 and GS-1 because of their relative importance and relevance to our presented case. The summary is presented in Table 3.

**Table 3:** Summary of case reports and case series included in the review ALT: alanine aminotransferase, APTT: activated partial thromboplastin time, AST: aspartate aminotransferase, ATT: anti-tuberculous therapy, BMAT: bone marrow aspiration and trephine biopsy, CBC: complete blood count, CMV: Cytomegalovirus, CNS: central nervous system, CT: computed tomography, CXR: chest X-ray, EEG: electroencephalography, F: female, GA: gestational age, gamma-GT: gamma-glutamyl transferase, GERD: gastrointestinal reflux disease, GS: Griscelli syndrome, HLH: hemophagocytic lymphohistiocytosis, HSCT: hematopoietic stem cell transplant, HTN: hypertension, IUD: intrauterine death, LDH: lactate dehydrogenase, M: male, MRI: magnetic resonance imaging, NF-1: neurofibromatosis 1, NK cell: natural killer cell, PBF: peripheral blood film, PT: prothrombin time, UTI: urinary tract infection

Sr. No.	Study ID	Demographics		History		Family history		Examination	Investigations	Diagnosis
		Area	Age/ Sex	Presenting complaints	Disease course	Cousin marriage	Cases	Organomegaly/ Neurological findings	CBC, PBF, BMAT, mutation, others	
1.	Panigrahi I et al. [2]	India	One year /F	Fever, milestones regression	The condition deteriorated with frequent seizures and died on day 14.	No	None	Dolichocephalic head. Tented upper lip. Organomegaly. Irritable, did not recognize her parents, visual issues.	Anemia on CBC. Leucocytes dropped during the stay. Normal lymphocyte subsets and NK cells on flow cytometry. The CD4/CD8 ratio was raised. Normal CSF analysis. Normal serum immunoglobulins. Normal BMAT. Brain MRI showed hyperintense lesions in periventricular, subcortical, and cerebellar areas.	GS-2 with HLH of CNS
2.	Singh A et al. [9]	India	Two and a half years /M	Progressive abdominal distension, pallor	Ear discharge for two months. Recurrent infections since the age of one year. Four blood transfusions.	No	Two siblings	Neurological signs and symptoms, and organomegaly	Pancytopenia on CBC. Ovalocytes and elliptocytes on PBF. Erythroid hyperplasia, megaloblastic erythropoiesis, depleted iron stores on BMAT. No increased hemophagocytic activity. Skin biopsy showed characteristic findings. Mutation in RAB27A.	GS-2
3.	Malhotra AK et al. [10]	India	One month /M	Pallor, recurrent fever, abdominal distension	Fever, cough, and abdominal distension at one month. Three blood transfusions. Treated for pneumonia and discharged. Two weeks later, emergency admission due to fever, cough, and abdominal distension. Died after four days due to septic shock.	No	One sibling. One IUD at three months GA.	Crepitations in both lungs. Organomegaly.	Anemia, neutropenia, and reduced platelets on CBC. Typical findings on skin biopsy. Erythroid hyperplasia on BMAT. Infiltrates in the right lung on CXR. On emergency admission, PBF showed atypical lymphocytes, and CXR showed infiltrates in both lungs. Postmortem biopsies showed increased hemophagocytic activity in bone marrow, liver, and spleen.	GS-2 with HLH.
4.	Ramasami S. [11]	India	Eight years /F	Fever, abdominal pain, and distension for 20 days	Underweight for age. History of red cell transfusion. The patient was taking ATT. She was given symptomatic treatment.	Yes	One sibling	Organomegaly	Anemia and leucopenia on CBC. Reverse albumin-to-globulin ratio, raised urea, and normal transaminases. CXR showed left lower lobe pneumonia. Skin biopsy showed characteristic findings.	GS-2
5.	Allesandra T et al. [12]	Romania	Six and a half years /F	Flu-like syndrome along with headache, drowsiness, dysarthria, and unsteadiness, followed by focal seizures and right hemiparesis.	Four months later, platelets were reduced, the liver and spleen were enlarged, and status epilepticus occurred. Developed HLH of CNS	No	None	Depressed at irritable, seizures. No organomegaly at presentation.	Normal CBC at presentation. MRI brain showed multiple hyperintense lesions in the brain and spinal cord. Cytopenias on CBC when HLH developed. CSF analysis showed raised proteins with a normal cell count. BMAT was normal. Brain biopsy showed hemophagocytic activity. A mutation in the RAB27A gene.	GS-2 with HLH
6.	Ghedda SA et al. [13]	Syria	Seven months /F	Loss of milestones	Ear infections. During admission, developed fever, seizures, respiratory distress syndrome, and died.	Yes	None	Organomegaly. Developmental regression.	Normal CBC at presentation. The head ultrasound was normal. CSF analysis showed viral meningitis. AST, ALT, PT, APTT raised, serum albumin reduced. Cytopenias on CBC when HLH developed.	GS-2 with HLH.
7.	Castaño‐ Jaramillo LM et al. [14]	Mexico	22 months /F	Loss of previously achieved milestones	Neurological issues and loss of milestones at 15 months. Acute visual loss and seizures at 22 months. HSCT at 27 months. HLH at 29 months. At 10 years, a second HSCT was proposed but was declined. She had elevated liver enzymes. The right eye remained blind.	Yes	Not written	Delayed milestones and loss of already achieved milestones. Organomegaly developed two months after HSCT.	Anemia on CBC. Elevated transaminases. CT brain showed cortical atrophy. The MRI brain showed white matter lesions. CSF showed increased proteins and mononuclear cells. Biochemistry showed elevated AST, ALT, LDH, gamma-GT, bilirubin, and triglycerides. Liver biopsies showed cholestasis. Normal BMAT. A mutation in RAB27A was not found. Diagnosis was made on impaired NK cell-mediated cytotoxicity by flow cytometry.	GS-2
8.	Khan I et al. [15]	Pakistan	27 years /M	Skin infection	Recurrent infections since childhood	Yes	One sibling	Normal	Normal CBC. Normal serum immunoglobulins. Typical skin biopsy findings.	GS-2
9.	Yeetong P et al. [16]	Thailand	Two years /M	Recurrent fever	Organo-megaly since nine months of age. At three years, ataxia and refusal to walk. Died at the age of four years despite HSCT.	No	None	Organomegaly	Pancytopenia on CBC. Normal or slightly raised serum immunoglobulin levels. CXR showed diffuse granular infiltration. MRI brain showed multiple bilateral lesions, including in the cerebellum. Increased hemophagocytosis on BMAT. Mutation in RAB27A.	GS-2 with HLH
10.	Nejad SE et al. [17]	Iran	One year /F	Albinism	Recurrent skin and respiratory infections	Yes	None	No organomegaly. Delayed milestones.	Anemia and leucopenia on CBC. Low serum immunoglobulins.	GS-2
11.	Emanuel PO et al. [18]	Hispanic	Two months /F	Albinism and neurological symptoms	GERD, recurrent respiratory infections, and leptomeningitis. During hospital stay, liver dysfunction, sepsis, and DIC developed. At 13 months, hypotonia, delayed milestones.	Yes	Uncles and grand-father from the side of the mother	Hypotonia, delayed milestones. Organ status not mentioned.	Pancytopenia on CBC. Skin biopsy showed prominent melanocytic melanin and absence of melanin in adjacent keratinocytes.	GS-2
12.	Kendir-Demirkol Y et al. [19]	Turkey	Nine months /F	Fever, abdominal distension	HSCT at one year. NF-1 at four years.	Yes	None	Neurological examination was normal. Organ status not mentioned.	Pancytopenia on CBC. Normal brain MRI at presentation. A mutation in the RAB27A gene. At 4 years, MRI brain and lumbar spine showed hyperintense lesions. A mutation in the NF1 gene.	GS-2 and NF-1
13.	Pehlivan UA et al. [20]	India	Five years /F	Quadriparesis, dysphagia, diplopia, urinary incontinence, and seizures	Died after four months due to pneumonia and sepsis.	Yes	None	Nystagmus, strabismus. Organ status not mentioned.	Normal CBC. The MRI brain showed hyperintense lesions. Brain biopsy showed increased histiocytic activity. Mutation in RAB27A.	GS-2 with isolated CNS disease
14.	Alsugair F et al. [21]	Saudi Arabia	One year /F	Albinism, recurrent chest infections	Recurrent chest infections at three- and six months of age. HSCT done. Developed acute lung injury as a complication of HSCT. Death due to AKI and sepsis.	Yes	1 sibling	Organomegaly	Pancytopenia on CBC. Brain MRI showed diffuse volume loss but no evidence of HLH. Follow-up MRI during hospital stay showed hyperintense lesions. Mutation in RAB27A.	GS-2
15.	Alsuheel AM et al. [22]	Saudi Arabia	Five months /F	Seizures, fever, abdominal distension	Seizures, HTN, CMV infection, UTI, severe pneumonia, death.	Yes	None	Seizures and organomegaly	Normal CBC. LDH, triglycerides, and ammonia were raised. CSF analysis was normal. Brain MRI showed severe cerebral edema with multiple brain parenchymal hematomas and dural sinus thrombosis. EEG showed encephalopathy.	GS-2 with dural sinus clot.
16.	Baothman AA et al. [23]	Saudi Arabia	45 days /M	Fever, irritability, decreased activity	HSCT done. Four years post-HSCT, he is alive and disease-free	No	One sibling	Organomegaly	Anemia and reduced platelet count on CBC. Normal CSF examination. Increased histiocytic activity on BMAT. Mutation in RAB27A.	GS-2 with HLH
17.	Fatima I et al. [24]	Pakistan	One and a half years /F	Drooling from the mouth for two days. Irritability and low-grade fever for 10 days.	GCS 2/10, seizures – day 3. She was given steroids with no alleviation of symptoms.	Not written	Not written	Mild organomegaly	Leucocytosis on CBC. Reactive lymphocytes on PBF. The MRI brain showed multiple hyperintense lesions.	GS-2 with HLH of CNS.
18.	Meschede IP et al. [25]	Brazil	Three and a half years /M	Fever and ataxia.	He was treated for cerebellitis. 2 months later, he developed pancytopenia, fever, organomegaly, and coma. He did not respond to treatment and died.	No	A twin sibling died of encephalitis at three years.	Quadriparesis and hypotonia	Normal CBC at presentation. Two months later, pancytopenia. CSF showed pleocytosis with prominence of mononuclear cells and increased proteins. FLAIR-MRI showed lesions in the right cerebral hemisphere and supratentorial white matter. A mutation in the RAB27A gene.	GS-2 with HLH.
19.	Usta T et al. [26]	Turkey	27 years /F	Fever for one month	Two similar episodes in the past.	No	None	Normal hair and skin color. Mild splenomegaly.	Pancytopenia on CBC. Phagocytosed RBCs on PBF. Raised transaminases, CRP, ferritin, and triglycerides. PET-CT showed generalized lymphadenopathy, increased bone marrow and spleen activity. A mutation in the RAB27A gene.	GS-2 with HLH.
20.	Bahrami A et al. [27]	Iran	Eight and a half months /M	Fever, purpura, vomiting	Was born through IVF. Prolonged icterus from Day 3 to Day 40 of life. Productive cough, eye infection, and enlarged liver and spleen a few days later. Hospital admissions one month later due to vomiting and two weeks after that due to diarrhea.	Not written	Not written	Normal hair and skin color.	Anemia, neutropenia, and reduced platelets on CBC. Normal BMAT. Normal Serum Immunoglobulins. Serum ferritin raised; triglycerides raised. A mutation in the RAB27A gene.	GS-2
21.	Masri A et al. [28]	Jordan	Six years /M	Fever, seizures, reduced activity, loss of appetite	Hospital admission for fever. Two weeks later, focal seizures and then generalized tonic-clonic seizures occurred. Deteriorated despite treatment. Developed aspiration pneumonia, sepsis, pancytopenia, and death.	Yes	Two siblings	Distended abdomen but no organomegaly. Generalized hypotonia, reduced power, deep tendon reflexes difficult to elicit.	Anemia on CBC. Normal PBF. Normal CSF analysis. Normal morphology BMAT with absent iron stores. Brain MRI showed hypersignal intensity in the periventricular area and the left thalamus.	GS-2 with HLH of CNS.
22.	Durrani S et al. [29]	Pakistan	Four months /M	Silver grey hair	No significant finding	Yes	Two siblings	Organomegaly	Pancytopenia on CBC. BMAT not done. Serum ferritin and triglycerides raised; fibrinogen low. Serum immunoglobulins were low. Mutation in RAB27A.	GS-2 with HLH
23.	Born AP et al. [30]	Denmark	13 years/F	Fever, lethargy, weight loss, dyspnoea.	Five weeks later, dyspnoea worsened. EBV isolated from plasma. Lost independent movement. Treated with steroids and etoposide and improved. HSCT after 18 weeks. Alive and stable.	Yes. The family's origin was from Pakistan.	None	Hyperlordosis, reduced muscle bulk and power, and peripheral edema. Organomegaly.	Anemia and leucopenia on CBC. Hypoproteinemia. Elevated liver enzymes. Myeloid metaplasia on BMAT. Biopsy of the right vastus lateralis showed myositis. 15 weeks after HSCT muscle biopsy showed no necrosis and inflammation. CK and myoglobin were raised. A mutation in the RAB27A gene.	GS-2
24.	Rajadhyax M et al. [31]	Asia	32 months /F	Drowsiness, unable to sit or stand	UTI two days ago.	Yes	None	Right sixth nerve palsy. Increased tone in both lower limbs and extensor plantar reflexes.	Normal CBC. Normal BMAT. CT head showed hydrocephalus. The MRI brain showed multiple contrast-enhancing lesions. Biopsy of cerebellar lesion showed hemophagocytic activity. A mutation in the RAB27A gene.	GS-2 with HLH of CNS.
25.	Al-Idrissi E et al. [32]	Saudi Arab	M	Partial albinism and repeated follow-ups since birth.	Premature birth due to rupture of membranes at 30 weeks of gestation, low birth weight, height, and head circumference. ARDS and mechanical ventilation at 58 days of age. Intra-ventricular cerebral bleed and hematoma at nine days. At age of 10 months, he had a developmental age of eight months.	Yes	None	Normal	Normal CBC. Cranial ultrasound showed cerebral bleed and hematoma. CT head at seven months showed no midline shift and no signs of recent bleed. A mutation in the MLPH gene.	GS-3
26.	Alonazi N et al. [33]	Saudi Arabia	Two months /M	Partial albinism since birth	No significant finding. Alive at 11 years.	No	None	Normal	Normal CBC. Normal serum immunoglobulin levels. NBT test and lymphocyte subset analysis were normal, with normal lymphocyte cytotoxic activities. A mutation in the MLPH gene.	GS-3
27.	Huang Q et al. [34]	China	32 years /M	Light colored hair and skin	No significant finding	No	None	Normal	Normal CBC. Normal serum immunoglobulin levels. Low platelet aggregation with ADP. Low fibrinogen. Normal brain MRI. A mutation in the MLPH gene.	GS-3
28.	Shah BJ et al. [35]	India	Nine years /M	Partial albinism	No significant finding	No	None	Normal	Normal CBC. Normal serum immunoglobulin levels. Typical findings on skin biopsy.	GS-3
29.	Wasif N et al. [36]	Pakistan	14 years /M	Partial albinism	No significant finding	Yes	One sibling	No organomegaly. Fundus examination showed hypopigmented spots in the macular area of the right eye, especially in the parafoveal area. Visual acuity was 6/9.	Normal CBC. Normal serum immunoglobulin levels. A mutation in the MLPH gene.	GS-3
			Seven years /F	Partial albinism	No significant finding	Yes	One sibling	Normal	Normal CBC. Normal serum immunoglobulin levels. A mutation in the MLPH gene	GS-3
30.	Kuldiji C et al. [37]	India	Nine months /M	Light colored hair and skin	No significant finding	Yes	None	Normal	Normal CBC. Skin biopsy showed regular distribution of melanin in the basal layer.	GS-3
			Nine months /F	Seizures	No significant finding	No	One sibling	Normal	Normal CBC. Examination of the fundus showed papilledema. Skin biopsy and neuroimaging were not done.	GS-1
			Two years /F	Light colored hair and skin	No significant finding	No	One sibling	Normal	Normal CBC.	GS-1
31.	Cagdas D et al. [38]	Turkey	Eight months /M	Absence of head control	Respiratory infection at four months of age. Hospital admission at 10 months for fever. At three years, milestones were delayed. Only sits. Does not speak.	No. Parents are from the same village.	None	Mental motor retardation, mild jerks.	Normal CBC. Brain MRI at the age of 10 months showed mild frontotemporal atrophy. Normal serum immunoglobulin levels. A mutation in the MYO5A gene.	GS-1
			Seven months /F	Absence of head control	Seizures since four months of age. At three years, seizures were partially controlled. Can not sit or speak.	Yes	None	Mental motor retardation and seizures	Normal CBC. Normal serum immunoglobulin levels. Brain MRI at one and a half years of age and brain auditory evoked potential examinations were normal. There was a delay in the visual evoked potentials, and the EEG showed disorganized background activity with frequent sharp wave discharges located in the left hemisphere. A mutation in the MYO5A gene.	GS-1
			Five years /F	Albinism	No significant finding	Yes	None	Normal	Normal CBC. Normal serum immunoglobulin levels. A mutation in the MLPH gene.	GS-3
			11 years /M	Albinism, an innocent cardiac murmur	Hospital admission for respiratory problems after birth.	Yes	None	Normal	Normal CBC. Normal serum immunoglobulin levels. A mutation in the MLPH gene.	GS-3
32.	Khorram E et al. [39]	Iran	Two months /M	Delayed milestones and albinism	Albinism since birth. Hypotonia at two months. Visual problems at 6 months. Seizure at 9 months. Diagnosis of GS at one year. Currently, five years old.	Yes	One sibling	Intellectual disability, speech absence, nystagmus, intermittent exotropia, vertical roving eye movement, sleep disturbance, feeding problems, short stature, growth retardation, UMN signs.	High voltage spikes on EEG. A mutation in the MYO5A gene.	GS-1
			One month /F	Delayed milestones and albinism.	Albinism since birth. Hypotonia at one month. Visual problems at five months. Currently, 18 months old.	Yes	One sibling	Delayed milestones, UMN signs, speech absence, did not react to sounds, lack of head control, sleep disturbance, and feeding problems. Syndactyly on both feet.	Elevated AST and ALT. A mutation in the MYO5A gene.	GS-1
33.	Abd Elmaksoud MS et al. [40]	Egypt	Seven months /M	Seizures	Head lag at three months. Could not sit at 6 months.	Yes	One sibling	Hypotonia, sitting with support, poor head control	Normal CSF analysis. The MRI brain showed a “bat wing” appearance. A mutation in the MYO5A gene.	GS-1
			Two months /F	Albinism since birth	No significant finding	Yes	One sibling	Hypotonia	Normal CSF analysis. The MRI brain showed subnormal brain development. A mutation in the MYO5A gene.	GS-1
			One year /M	Delayed milestones	No significant finding	Yes	One cousin	Hypotonia, sitting with support, spoke few words only	Normal CSF analysis. MRI brain showed isolated cerebellar atrophy. Mutation in MYO5A gene.	GS-1

Case discussion and literature review of Griscelli syndrome type 2

This part of the review includes 24 case reports [2,9-31]. Some of the reference numbers with specific case report findings are cited in the text. Our patient was a male who presented in the first decade of his life. This is consistent with many case reports in the literature where patients' first encounter with the physician is during the early years of life. The youngest patient in our case reports of GS-2 was 1 month old, and the oldest one was 27 years old. We had 14 female patients and 10 male patients in our collected data. Our patient presented with a history of recurrent drops in hemoglobin and pallor. Singh A et al. and Malhotra AK et al. also reported the same presenting complaints in their case reports [9,10]. Other symptoms driving parents to doctors in our data include fever in 11 patients, neurological issues in 12 patients, abdominal distension in 4 patients, and albinism in 2 patients. Past history of recurrent infections, as seen in our patients, is also strongly supported by the literature. Thirteen out of 24 case reports had a past history of recurrent infections. Another finding of significance in the past histories of patients is a history of single or multiple transfusions reported by three case reports [9-11]. Our patient also had a history of transfusions due to recurrent drops in hemoglobin. Most of the patients in our data were products of consanguineous marriage. Our patient's parents, however, were not cousins. Eight cases are included in our review where GS-2 has been diagnosed in children whose parents were not cousins. On examination, our patient was developmentally normal with no neurological signs and symptoms and had hepatosplenomegaly and pallor. In our data, 12 out of 24 patients had neurological symptoms. Organomegaly was seen in 16 patients. Six patients had normal livers and spleens. The status of the rest of the patients was not mentioned. Cytopenias are a common finding in GS-2. Our patient had pancytopenia. However, in the literature, we found combinations of cytopenias, and some patients had normal CBC findings as well. Two patients had normal CBC at presentation but developed cytopenias as the disease progressed and HLH developed [12,13]. HLH has been widely reported in the context of GS-2. Twelve patients in our reviewed case reports had HLH. Eight of these patients had increased hemophagocytic activity in the CNS. Our patient had a normal neurological examination showing no involvement of the CNS. Characteristic hair shaft findings on microscopy were found in all patients presenting with GS-2. Our patient also had similar hair shaft findings. Among the patients whose mutation analysis was carried out, only one was found to be negative for the RAB27A mutation. His diagnosis was confirmed by NK cell activity on flow cytometry [14].

There were some interesting case reports as well. One of them with negative mutation analysis has already been discussed. One patient developed neurofibromatosis type 1 [19]. Another interesting case presented with isolated CNS disease and no other findings [20]. There is one case report of a patient who developed cerebral dural sinus thrombosis [22]. One of the cases presented with normal skin and hair color. The rest of the history and examination were consistent with GS-2 [26].

GS has been reported as an autosomal recessive disease in the literature. However, in the case of our patient, only males of this family were affected. The patient’s two sisters were alive and healthy. He and his younger brother were both affected. On the maternal side of his family, one family also suffers from the same pattern. His two maternal uncles had the same appearance and symptoms, while his maternal aunts were healthy. This pattern might point toward an X-linked pattern of inheritance. Due to the death of the patient a few days after he was diagnosed, the death of all the affected cases in his family, and financial constraints, we could not offer genetic testing. We propose that this pattern of inheritance must be vigilantly looked for in other affected families as well. The disease might follow more than one pattern of inheritance.

Griscelli syndrome type 3

A total of nine patients in our selected studies had GS-3. Two of them were females and seven were males. The youngest patient was being followed since birth [32]. The highest age at presentation was 32 years [34]. All nine patients had albinism as the presenting complaint. Six patients were products of cousin marriage, and only two had affected siblings in their families. CBC of all patients was normal. Serum immunoglobulins were checked in seven cases and were normal in all of them. One patient had abnormal platelet aggregation with adenosine diphosphate (ADP) and reduced fibrinogen levels [34]. Cardiac murmur and abnormal findings on the examination of the fundus were seen in one patient each [36,38]. Of special interest was the patient reported by Al-Idrissi et al. This case has already been cited as the youngest patient at presentation. His mother went through a premature rupture of membranes and gave birth to him at 30 weeks of gestation. Since birth, he had albinism, and he suffered from recurrent infections afterward. He also had cerebral bleeds, but they were found to be unrelated to GS [32]. Mutation analysis was done in seven patients, and all of them were found to be positive for the MLPH gene mutation.

Griscelli syndrome type 1

A total of nine cases were included in the review. Four of them were males and five were females. The youngest age at presentation was one month, and the oldest was two years. Two patients presented with albinism and seven with neurological symptoms. Seven patients had abnormal neurological findings on examination as well. CBC was normal in all the cases. Serum immunoglobulins were checked in two patients, and results were normal [39]. One patient had a normal MRI brain, but the EEG was found to be abnormal [38]. An abnormal MRI brain was found in four patients. Subnormal brain development, cerebellar atrophy, frontotemporal atrophy, and bat wing appearance were some of the abnormalities noted [38,40]. Mutation analysis was carried out in seven patients. All of them were positive for the MYO5A gene.

Approach to diagnosis

Next, we present an approach to albinism from a hematologist's perspective. Many articles focus on the approach to albinism. We, however, feel the need to propose an approach a hematologist would follow while approaching a patient with albinism. This would include history, examination, and investigations.

The history of recurrent infections points toward immunodeficiency. CHS, HPS, and GS all three can have immunodeficiency [41]. However, it is more commonly seen with CHS and GS-2. A history of platelet-type bleeding or easy bruising points toward platelet count or function abnormalities. Bleeding problems are most pronounced in HPS, but CHS and GS can also present with bleeding issues when the platelet count drops, particularly in the setting of HLH. A patient suffering from HPS may also have respiratory symptoms given its association with pulmonary fibrosis [42]. Diarrhea resulting from granulomatous colitis can also be among the presenting complaints of HPS [43]. Birth and developmental history with special reference to developmental delays or regression should be taken. Any neurological symptoms can be due to primary neurological issues in GS-1 or secondary to HLH in GS-2. A history of consanguinity and similar cases in the family should be carefully taken. Family origin is important because patients with HPS are mostly from Puerto Rico.

Physical examination should start with a general physical examination. Bruises and bleeding should be noted. Abdominal examination should be done for organomegaly and the status of lymph nodes. Organomegaly is mostly seen in HLH, and patients with infection may present with lymphadenopathy. A complete systemic examination should follow. Patients with GS-1 and GS-2 usually present with CNS abnormalities as discussed in the preceding paragraph. Abnormalities in the respiratory examination may point towards HPS.

For a hematologist, investigations start with CBC and PBF. Peripheral blood counts can present in a number of ways in cases of CHS. WBC counts may be normal or may show neutropenia or pancytopenia in the setting of HLH. A characteristic finding in PBF in CHS is giant granules in the granulocytes and lymphocytes [44]. In GS-1 and GS-3, CBC and PBF are normal. In GS-2, cytopenias may be there due to its association with HLH. Reactive lymphocytes in the PBF are not an uncommon finding in GS-2. In HPS, CBC and PBF are normal, or thrombocytopenia may be present [45].

Light microscopy of hair is an easy and readily doable test. It is not diagnostic in HPS, but in CHS and GS, it provides conclusive information. In GS, the hair shafts show large clumps of melanin that are irregularly placed in the medulla with a clear cortex [46]. In CHS, these pigment clumps are large but smaller than in GS and regularly spaced in the medulla [47].

The next non-invasive test that can give powerful insight into the diagnosis is platelet aggregation. In the settings where this is not available, bleeding time (BT) can be performed. BT is prolonged in CHS and HPS. In GS, it is prolonged in cases with thrombocytopenia. There can be issues with dense granules in cases of GS-2 as well. Platelet aggregation studies show storage pool defect in cases of CHS and HPS, while it can be normal in GS or may show dense granule defect [48,49]. These tests, however, have certain prerequisites to be met for reliable results. The most important of them is the patient's platelet count. Patients with very low platelet counts are not suitable to undergo BT and platelet function tests [50].

Bone marrow aspirate in case of CHS shows giant granules in the myeloid lineage and lymphocytes. In the accelerated phase of CHS, hemophagocytosis is seen in the bone marrow. In GS-1 and GS-3, bone marrow is usually normal. In GS-2, prominent hemophagocytosis is seen.

**Figure 6:**
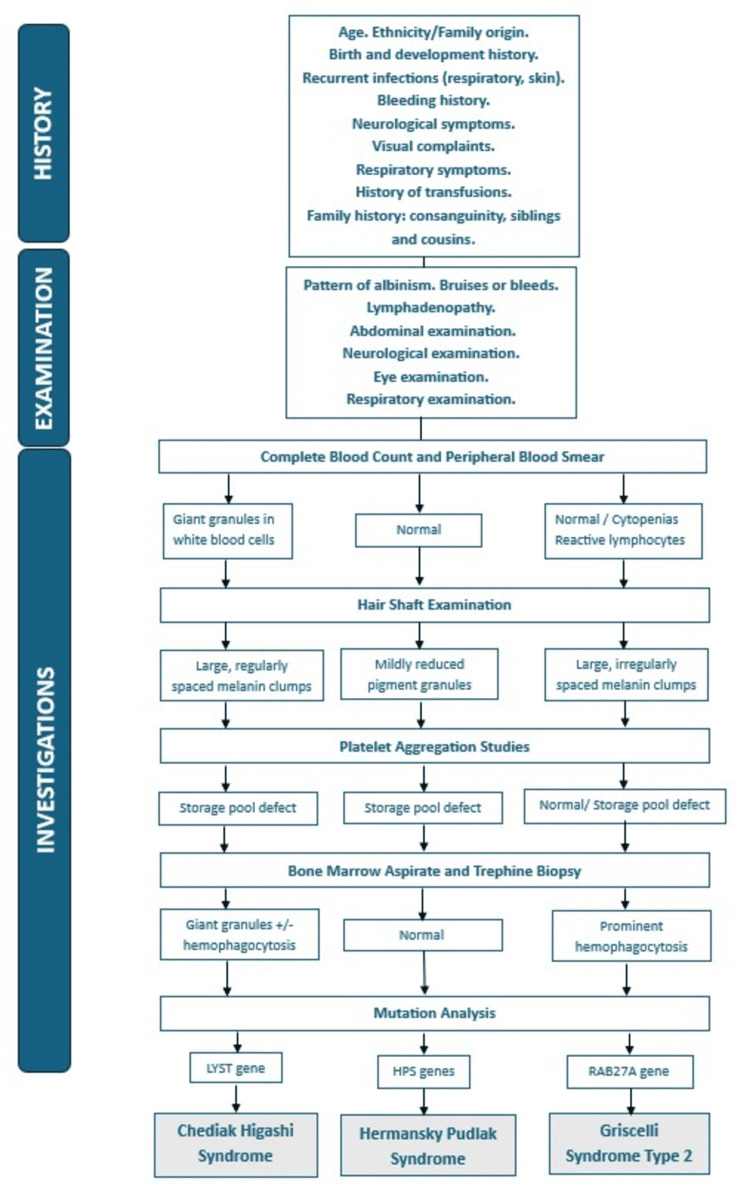
Diagnostic approach to hematological disorders associated with albinism This figure is the authors' own creation and is not reproduced from published literature. * Bleeding time and platelet aggregation studies can be skipped if the pre-requisites for the tests are not met or the facility is not available. The diagnosis can be reliably made without this investigation as well. A storage pool-like defect is seen in Griscelli syndrome type 2, and platelet aggregation studies show normal results in cases of Griscelli syndrome type 1 and type 3.

Mutation analysis remains the confirmatory test. In resource-constrained countries like ours, most of the patients cannot afford it, and most settings do not offer mutation analysis as well. The culprit genes in cases of CHS and HPS are the LYST gene and the HPS gene, respectively. In GS-1, GS-2, and GS-3, the mutations are found in the MYO5A, RAB27A, and MLPH genes, respectively. The simplified approach is given in Figure 6.

**Figure 7:**
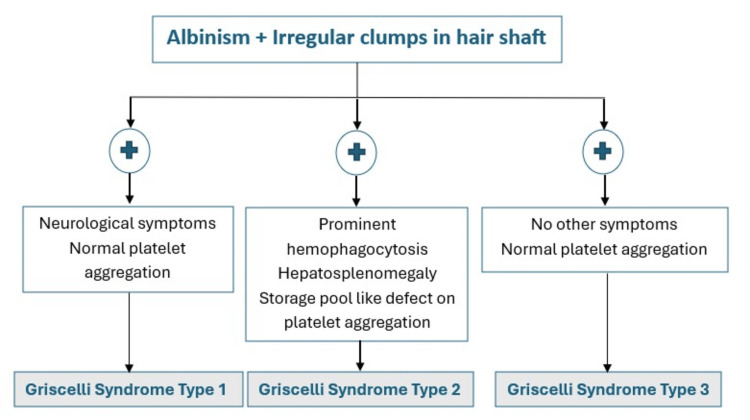
Types of Griscelli syndrome This figure is the authors' own creation and is not reproduced from published literature.

If we integrate platelet aggregation findings in the diagnosis, we can say that along with albinism and characteristic hair shaft findings, neurological abnormalities and normal platelet function are seen in GS-1; immunodeficiency, HLH, and storage pool-like defects are seen in GS-2, and in GS-3, no other abnormality is seen in addition to albinism and hair shaft findings. We have summarized this information in Figure 7.

## Conclusions

Griscelli syndrome is a rare disorder, and a great deal of clinical experience is required for the suspicion and diagnosis. Our case shows typical findings of type 2 Griscelli syndrome, but at the same time, raises concerns about a pattern of inheritance other than autosomal recessive. Partial albinism, often syndromic, is approached and treated as a medical issue, making the propensity of missing this rare diagnosis even higher because of a lack of hematologists’ input. We have, therefore, proposed an algorithm for a hematological approach to disorders associated with albinism as well.
